# Supplementary material for: Parathyroid Hormone Induces Human Valvular Endothelial Cells Dysfunction That Impacts the Osteogenic Phenotype of Valvular Interstitial Cells
Source: Int J Mol Sci. 2022 Mar 29;23(7):3776. doi: 10.3390/ijms23073776 (PMC8998852; doi:10.3390/ijms23073776)
Supplement: Supplementary file 1 [file ijms-23-03776-s001.zip › ijms-1638940-supplementary.pdf]

Supplementary methods

Glucose quantification assay

Glucose levels in the condition media from VEC exposed to PTH for 48h or 7 days were quantified using Glucose, GOD-PAP kit (DIALAB), according to manufacturer instructions.

Supplementary figures

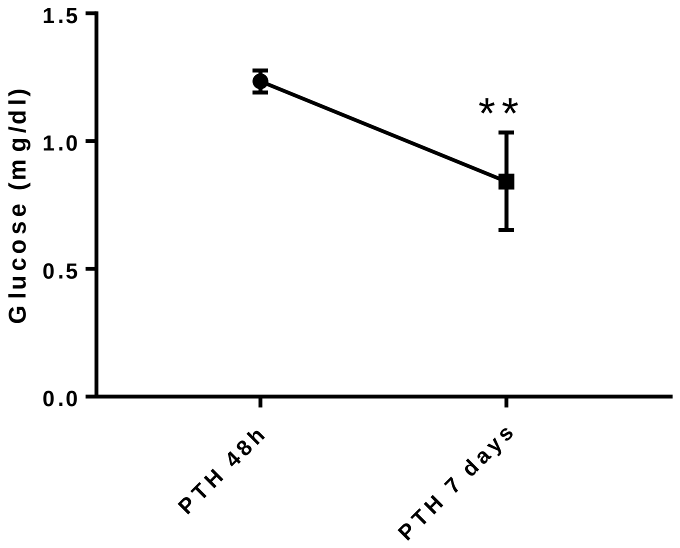

Supplementary figure S1. *Evaluation of glucose levels in conditioned media from VEC exposed to PTH for 48h or 7 days. Conditioned media was analyzed for glucose levels using a glucose detection kit, **p <0.01.*

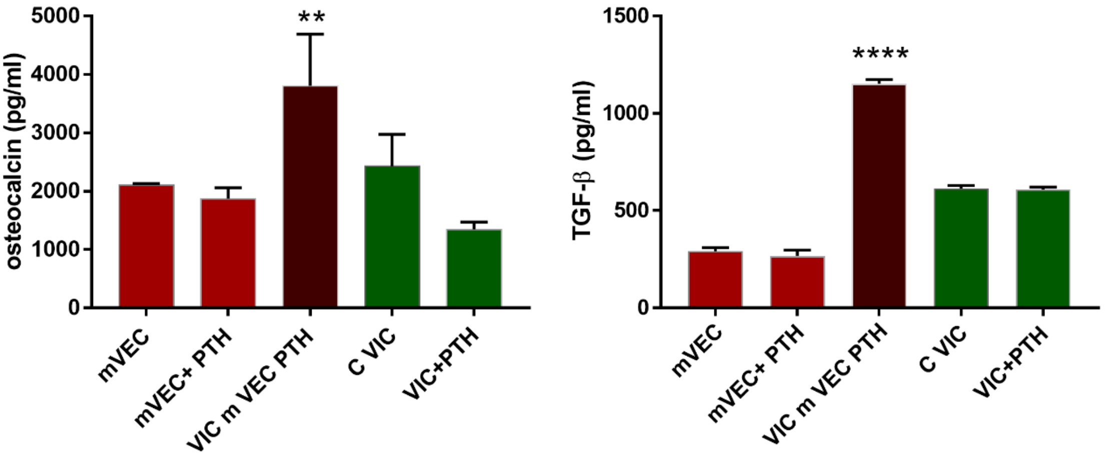

Supplementary figure S2. *Osteocalcin and TGF-β levels released by VEC or VIC in different experimental conditions:* ***mVEC*** *– CM from control VEC,* ***mVEC+PTH*** *- CM from VEC exposed to PTH,* ***VIC mVEC+PTH*** *- VIC treated with conditioned media from VEC exposed to PTH,* ***C VIC*** *- CM from control VIC,* ***VIC+PTH*** *– CM from VIC exposed to PTH, measured by ELISA. Note that CM from VEC or VEC+PTH or VIC exposed directly to PTH does not exhibit increased levels of osteocalcin or TGF-β. n = 3,* **p < *.01,* ****p < *.0001.*

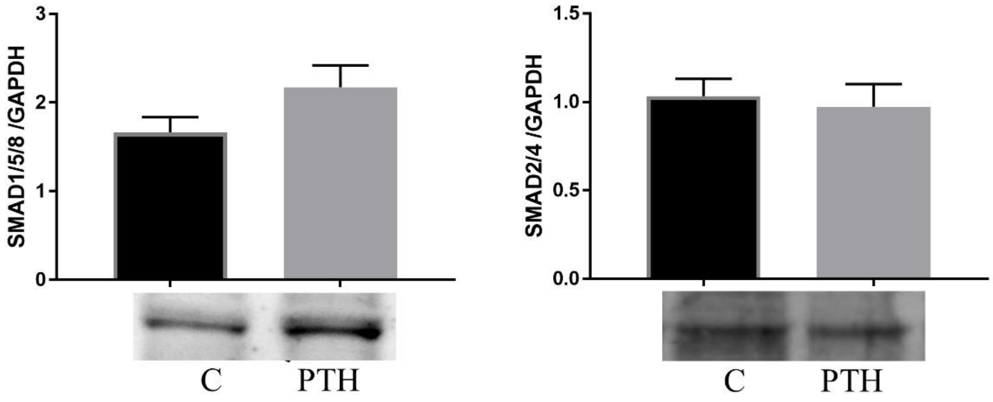

Supplementary figure S3. *Protein expression of SMADs expressed by VEC exposed to PTH. Quantification of protein expression of SMAD1/5/8 and SMAD2/4 in VEC as determined by Western blot. (F) Representative Western Blot images for investigated molecules are presented.*

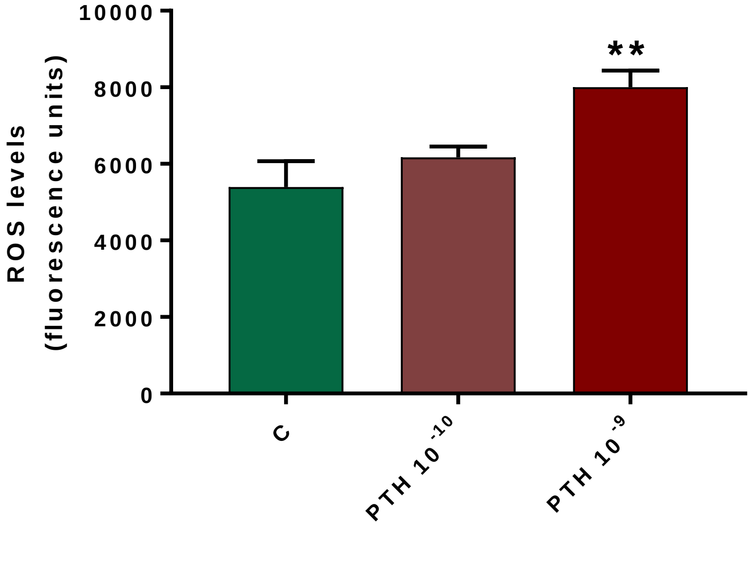

Supplementary figure S4. Evaluation of ROS production in response to two different concentrations of PTH. *VEC were treated with PTH (10^{-10}M, 10^{-9}M) and the total amount of ROS was determined incubating the cells with the fluorescent probe DCFDA. The ROS levels were expressed as relative fluorescence units (**p < 0.01 vs. C). The image is the mean of three independent experiments.*
